# Factors influencing provider deviation from national HIV and nutritional guidelines for HIV-exposed children in western Kenya: a qualitative study

**DOI:** 10.1186/s12913-024-11942-9

**Published:** 2024-11-26

**Authors:** Megan M. Coe, Emily Yoshioka, Damaris Odhiambo, Mary Masheti, Phlona Amam, Julius Nyaoke, Emmanuel Oduor, Marline Serede, Agnes Ndirangu, Benson Singa, Arianna Rubin Means

**Affiliations:** 1 School of Nursing, University of Washington, Seattle, USA; 2 Department of Global Health, University of Washington, Seattle, USA; 3 KEMRI-UW, Migori, Kenya; 4 KEMRI-UW, Homa Bay, Kenya; 5 Centre for Clinical Research, Kenya Medical Research Institute, Nairobi, Kenya

**Keywords:** HIV Exposed Infants, Nutrition, Qualitative Research, Kenya, Theoretical Domains Framework, Service integration, Guidelines

## Abstract

**Background:**

Malnutrition and HIV interact in a vicious cycle for HIV-exposed infants (HEIs), increasing vulnerability and the severity of each condition and contributing to poor health outcomes. We identified multi-level factors influencing provider adherence to Kenyan HIV and nutrition guidelines for HEIs.

**Methods:**

We conducted six focus group discussions and seven in-depth interviews using a semi-structured question guide. Participants were selected through purposive maximum variation sampling of health workers involved in maternal and child health services and outpatient nutrition programs at two facilities in western Kenya. Data collection and analysis were guided by the Theoretical Domains Framework (TDF). Transcripts were coded by two primary coders using both deductive and inductive thematic analysis.

**Results:**

TDF domains that drove guideline adherence included: environmental context and resources, beliefs about capabilities, and social influences. While participants praised attempts to integrate HIV and nutritional services through teamwork and service colocation, challenges in the successful referral of patients between services persisted. Participants described siloed HIV and nutrition-related knowledge across staff, leading to missed or delayed care if certain providers were unavailable. Participants emphasized understaffing as a major contributor to gaps in care. Inconsistent material resource availability also disrupted linkages between HIV and nutrition services for patients. While participants frequently expressed high intention and internal motivation to link children between services, they described minimal structured supervision or positive reinforcement from supervisors and feeling demoralized when resource constraints interfered with care provision. Lastly, participants described patient-level factors that made it challenging for families to seek or remain in care, including poverty and HIV and malnutrition-related stigma. Participants made several recommendations, including training multiple cadres in the fundamentals of both HIV and nutritional care to address siloed services and understaffing.

**Conclusions:**

This study details the factors that facilitate or hinder health workers as they implement national guidelines and link HEIs between HIV and nutritional services, including the impact of physical integration of service sites, human and material resource constraints, and health worker motivation. Future interventions can address these challenges by expanding access to needed resources, task sharing, and testing implementation strategies that increase the efficiency of service delivery to improve linkages in care for vulnerable infants.

**Supplementary Information:**

The online version contains supplementary material available at 10.1186/s12913-024-11942-9.

## Introduction

HIV-exposed infants (HEIs), who are born to mothers living with HIV, are uniquely vulnerable to poor health outcomes [1, 2]. In Kenya, there were 52,000 pregnant women with HIV in 2022, and while there were 4,500 new HIV infections among children, all HEIs require heightened attention [3]. HEIs have mortality rates two to four times as high as HIV-unexposed children and reduced weight and length growth compared to unexposed children [4–7]. HIV exposure can impact the growth and development of HEIs through multiple pathways, including direct exposure to infection in utero, suboptimal breastfeeding, and maternal health status [8]. Malnutrition is an underlying or immediate cause of nearly half of child deaths [9, 10]. Both malnutrition and HIV exposure are associated with changes in the immune system that increase morbidity from common childhood infectious diseases [11, 12]. Therefore, it is important to assume a coordinated approach to addressing these conditions together.

Kenyan national HIV and nutrition guidelines call for all HEIs to receive HIV prophylaxis until six weeks after cessation of breastfeeding and for their growth and feeding to be assessed at every clinic visit for early detection and treatment of malnutrition [13, 14]. Similarly, for malnourished children whose HIV status is negative or unknown, guidelines recommend they receive immediate HIV testing given that acute malnutrition may be an early indication of HIV infection [15]. Operationalizing these guidelines often requires linking patients between several providers, programs, and clinics, such as maternal child health (MCH) clinics that provide services including immunization and growth monitoring, prevention of mother-to-child transmission (PMTCT) clinics that are specific to HIV prevention, and nutrition clinics. While evidence suggests that there are multiple factors affecting the linkage between HIV care for mothers and their infants, there is a dearth of evidence about factors influencing linkages between HIV and nutritional care [16–18].

Engaging frontline health workers is an effective way of identifying solutions to context-specific implementation challenges, such as the uptake of guidelines [19]. The objective of this qualitative study was to identify the patient-, provider-, and facility-level factors that influence adherence to HIV and nutritional guidelines among health workers caring for HEIs in two facilities in western Kenya. Identifying these factors directly from frontline health workers is critical to developing implementation strategies that are well-matched to the barriers at hand.

## Methods

### Study setting

We collected qualitative data at two facilities in western Kenya, Homa Bay County Teaching and Referral Hospital (HBCTRH) and Migori County Referral Hospital (MCRH), from June to July 2021. In Homa Bay and Migori counties, 1.8% and 2.4% of children under five are classified as wasted (weight for height Z-score < −2), and 12.5% and 14.8% of children under five are classified as stunted (height for age Z-score < −2), respectively [20]. The HIV prevalence in 2021 among females aged 15–49 was estimated to be 19.2% in Homa Bay and 15.5% in Migori counties [21]. The county-level HIV incidence per 1000 children aged 0–4 years in 2023 was estimated to be 1.5 in Homa Bay and 1.4 in Migori, approximately triple the national level (0.5) [21]. At both facilities, HIV and nutrition services (including growth monitoring and promotion) for children are colocated within MCH clinic areas. According to Kenyan national guidelines, HEIs with severe acute malnutrition should be provided with institutional nutritional management and supported with ready-to-use therapeutic food [14, 15]. Meanwhile, children with moderate acute malnutrition should be enrolled in a supplemental feeding program, however, the recommended supplements were frequently out of stock during the study period [14, 15].

### Sampling

This study included in-depth interviews (IDIs) and focus group discussions (FGDs) with health workers involved in caring for HEIs. Health workers were eligible to participate in this study if they were involved in MCH, HIV, nutrition, and/or pediatric services (there were no exclusion criteria). We aimed to conduct a minimum of six FGDs with at least 30 health workers. Health workers were recruited using a purposive maximum variation sampling approach to ensure demographic representation across years of practice (< 5 years, 5–10 years, 10 + years of practice), cadre (e.g., doctor, nurse, nutritionist), sex, and age. FGDs ranged in size from 5–10 participants. Health workers participated in either an IDI or FGD.

### Data collection

A trained Kenyan social scientist with extensive experience engaging health facility staff conducted all IDIs and FGDs. We sought to make all participants feel free to voice their opinions by selecting a facilitator who did not live or work near the health facilities, but who built rapport and encouraged balanced participation in FGDs. While FGDs were the primary data collection method, IDIs were conducted as needed to maintain flexibility to participant schedules, address concerns regarding group activity during COVID-19, and facilitate more open discussions among staff by separating participants holding supervisory roles (e.g., nurse in charge). We obtained written informed consent before data collection, with consent forms available in four languages. English is the primary language of the healthcare system in Kenya and all participants chose to consent in English. The facilitator was fluent in multiple languages spoken in Kenya and participants were free to use other languages when needed to express themselves, however this rarely occurred. IDIs and FGDs were conducted in private spaces and audio recorded. The facilitator used a semi-structured question guide developed for this study and informed by the Theoretical Domains Framework (TDF) (Appendix 1). The TDF was selected for this study as it offers a taxonomy of factors that influence health worker behaviors and the use of evidence-based recommendations [22]. The TDF includes 14 domains and 84 theoretical constructs that characterize cognitive, affective, social, and environmental determinants of behavior [22]. We selected 12 domains from the TDF to inform question guide development based on relevance to the research question and included additional questions regarding health workers’ ideas for interventions that could ameliorate the challenges they face in adhering to guidelines in practice. FGDs ranged in length from 60 to 90 minutes, and IDIs ranged from 30 to 60 minutes. After each IDI or FGD, the social scientist completed a standardized debrief form to document key messages and reflections. Each participant received 600 Kenyan shillings (approximately $5.50 USD) for their time.

### Data analysis

Audio recordings were transcribed verbatim and any statements made in other languages were translated to English. An individual who did not perform the original transcription completed quality assurance, spot-checking two one-minute random segments of each transcript. Thereafter transcripts were uploaded to Dedoose [23]. We used a primarily deductive approach and developed a codebook *a priori* using the TDF as a guide (Appendix 2) [24–26]. Two independent coders at the University of Washington conducted thematic coding and shared primary and secondary coding responsibilities. Transcripts were equally assigned between coders and both coders acted as primary coders on their assigned transcripts, independently reading and coding each transcript. Then, both coders validated the other coder’s transcripts by reviewing coded transcripts in full and recording instances of disagreement with the code application. The coders met weekly to resolve any discrepancies between codes as necessary, and the codebook was iteratively updated as necessary to refine code definitions or to include inductive codes added and agreed upon [27]. When consensus could not be reached, the coded statement went to a third coder who acted as a tiebreaker.

Case memos were created for each health facility to describe and compare emerging themes [28]. For each emerging theme, the memos detailed (1) what TDF domains/constructs were influential, (2) if the determinant of HIV and nutritional care manifested as a barrier or facilitator, (3) supportive coded statements from the relevant data, and (4) a summary of how the emerging theme related to other noted emerging themes.

Case memos were discussed among study personnel and emerging themes were refined and finalized. In July 2022, a member-checking process was conducted to validate the findings [29]. Participants were selected from health workers who worked at the health facility for longer than six months and worked in HIV, nutrition, MCH, or hospital leadership. The final themes were presented to participants (*n* = 8 at HBCTRH, *n* = 7 at MCRH) and they were asked to consider if the themes fully captured the main factors influencing linkages between HIV and nutrition services or if any important topics were missing.

## Results

A total of 55 health workers participated in six FGDs (*n* = 48 participants) and seven IDIs (*n* = 7 participants). Among participants, 51% were aged 30–39, 82% were female, and 29% had each 1–4 and 5–9 years of experience (Table 1). Overall, 36% of participants were nurses, 25% were peer educators/mentors, 13% were clinical officers, 13% were nutritionists, 9% were counselors (HIV testing and adherence), and 4% were health workers with other roles (Table 1). Seven themes emerged as primary factors influencing adherence to HIV and nutritional care for HIV-exposed children: co-location of services, siloed knowledge, understaffing at health facilities, erratic resource availability at a facility level, low health worker confidence, poverty that affects family care seeking, and HIV and malnutrition-related stigma. TDF domains that drove themes included knowledge, skills, social/professional role and identity, beliefs about consequences, reinforcement, and intentions; environmental context and resources, beliefs about capabilities, and social influences were particularly influential.

**Table 1:** Demographic characteristics of study participants (*N* = 55)

Characteristic	*n* (%)
**Age**	
20–29	14 (25)
30–39	28 (51)
40–49	11 (20)
50 +	2 (4)
**Sex**	
Female	45 (82)
Male	10 (18)
**Years of experience**	
< 1 year	7 (13)
1–4 years	16 (29)
5–9 years	16 (29)
10–14 years	10 (18)
15 + years	6 (11)
**Role**	
Nurse	20 (36)
Peer educator/mentor	14 (25)
Clinical officer	7 (13)
Nutritionist	7 (13)
Counselor (HIV testing and adherence)	5 (9)
Other	2 (4)

### Colocation of services has improved HIV and nutrition service integration, but services are not fully integrated

Respondents in both facilities frequently praised efforts to integrate services and staff across HIV and nutritional care, primarily through the colocation of services. Health workers described the MCH as a *“one-stop shop”* (Facility 1 IDI #4 and Facility 1 FGD #2) where HIV and nutritional care is provided under *“one roof”* (Facility 2 FGD #3, Facility 1 FGD #1, and Facility 1 IDI #1). One respondent explained that,

*[At MCH, HEIs] have their clinicians, they have their lab, they have their nutritionist, they have their nurses, and they have their pharmacy there, they also have counselors there, so it is like a supermarket…So, they come in and finish everything there.* (Facility 2 IDI #1)

Health workers provided several examples of varying levels of coordination between staff working to facilitate linkages between HIV and nutrition services. One respondent remarked that *“because of the teamwork you will realize that [when] a nurse working at the PMTCT gets a child who needs nutritional services…some of them will stop doing whatever they are doing…[and] will physically pick the patient and bring the patient to the nutrition clinic”* (Facility 1 IDI #1). Another respondent explained at MCH, *“people work as a team, the nurses work together with the clinicians and the nutritionist…[I]t is not one person’s thing”* (Facility 2 IDI #1). While teamwork was highlighted, one participant described tension between HIV and nutrition services staff when services are missed for a patient:

*[T]rust me at times [HIV and nutrition services staff] could even fight because somebody comes to the clinic alongside the client and the MCH booklet [medical record], and they ask you why you missed some aspects. So, you will feel like she is giving you a lecture, but at the back of her mind it is something that you didn’t do, so it is a priority.* (Facility 2 IDI #3)

Despite the successful colocation of HIV and nutritional services, health workers often noted that patients do not successfully transition between PMTCT services and nutrition clinics due to several reasons, including limited signage and lack of personnel to escort families from one service to another. Health workers proposed several solutions to address this challenge, including escorting patients to nutrition clinics. Some health workers noted they already try to do this, but understaffing makes it challenging to consistently implement. Several respondents suggested improving directional signage to the nutrition clinic or staffing a central receptionist to direct patients moving from one service to another. Finally, one health worker suggested strengthening the internal referral system.

*[W]e can have something like an indicator that will show…we referred this number of kids to the nutrition clinic and this number reached the nutrition clinic so that we can be sure…the children we are referring to nutrition clinic actually reach there, and they actually receive services.* (Facility 1 FGD #1)

### Gaps in HIV and nutrition knowledge and skills prevent service linkages

Health workers described ongoing challenges driven by siloed knowledge and skills. Respondents said this *“knowledge gap”* created missed opportunities for providing HEIs nutritional treatment and counseling, as well as providing HIV-related services to undernourished children (Facility 1 IDI #4). As an example, guidelines for breastfeeding amongst mothers living with HIV were discussed by several participants as an intersecting topic that health workers were not aligned on.

*The other challenge we are having in delivering the nutrition care is about conflicting nutrition information from the health staff…[W]e are getting some mothers stopping breastfeeding at six months, that early. Some even stop it before reaching six months and that is too dangerous because our main aim is to boost that immunity for these babies…and when you ask these mothers they tell you, ‘I was told by the doctor’ and now we don’t know who delivered that information.* (Facility 1 IDI #2)

Similarly, it was noted that the Ministry of Health (MOH)-hired staff and staff hired by partner non-governmental organizations (NGOs) have differential knowledge of HIV and nutritional care. Two participants described this division.

*Like for us NGO [staff] we are expected to know [laughter]. So now when there are new updates, they print SOPs for us then we stick on the wall…* (Facility 1 FGD #3)

*MCH staff are segregated, we have [NGO] staff and we have the ministry staff…[NGO staff] are so equipped with HIV related issues and these other ones know nothing about it. So you find the other staff have no knowledge and you find there is some gap in case such a patient comes and these other staff for the government are the ones that are there, they will not know how to handle them that much as a[n] [NGO] staff would.* (Facility 1 IDI #4)

Siloed HIV and nutritional knowledge amongst staff is most evident when a nutritionist is unavailable. One health worker explained: *“[S]ometimes you might have everything, but the nutritionist is not there. And you have F75, you have F100 [therapeutic milks for treating severe acute malnutrition], you have everything, but you don’t even know how to start administering”* (Facility 2 FGD #1). Due to the shortage of health workers with topical expertise, several respondents proposed increasing the training of staff across HIV and nutritional care. This was particularly relevant for non-nutritional staff.

*I think all health workers from in charge officer, to the clinician, to the nurse in the ward, we need to be given that nutritional knowledge so that when you see a child, I don’t have to run to [the nutritionist] to assess that child. I should be able to assess that child there and then. I should be able to take weight, I should be able to take height. I should be able to take [mid upper arm circumference] and calculate [body mass index] without looking for the nutritionist who is so overwhelmed. *(Facility 2 FGD #2)

Health workers suggested that non-nutritional staff be trained on growth monitoring and nutrition assessments, including anthropometric measurements and prescription of nutritional treatment. One health worker noted the importance of *“continuous”* and *“holistic”* capacity building for HIV and nutritional treatment and care (Facility 2 IDI #1).

### Understaffing is a major contributor to gaps in HIV and nutritional treatment and care

Understaffing across services was highlighted as a major contributor to gaps in the provision and linkage of HIV and nutritional treatment and care. Due to understaffing, health workers felt *“fatigue[d]”* (Facility 2 FGD #2) and *“overwhelmed”* (Facility 2 FGD #2, Facility 1 IDI #2). For some patients, certain services might be omitted completely during a visit due to workload and understaffing. For example, respondents said that some tasks, like preparing food supplements, might be missed due to understaffing.

*[I]n the ward we have one nutritionist she's now on leave, these children are suffering… [Y]esterday we had an admission…she has not been assessed, no therapeutic food…started for this child. So, it is like they have come to the ward to be helped and we haven’t even started them on that therapeutic feeds.* (Facility 2 FGD #1)

Understaffing may also contribute to long queues, which health workers described as having multiple consequences.

*[A] mother can come to the PMTCT and you try to link her to the nutritionist, but when she gets there will depend on when she completes her procedures at the PMTCT. Maybe she came late and found the queue very long and she has queued for a long time. And now again you want her to go to the nutrition, when you refer her there she will go but if she finds the doctor is not there or the queue is long her probability of disappearing is very high because she feels she already gotten her medication and that is what she had come for.* (Facility 1 IDI #3)

Shortages of nutritionists were a major concern for both HIV and nutritional staff. One respondent stated,

*[T]he greatest challenge is the human resource because as we speak in the MCH where the services are offered we only have one nutritionist…[B]ecause this nutritionist doesn’t only offer nutrition services to infants that are HIV-exposed, he offers services to all the children who come there. So, at times you realize that this mother has come, he has other patients to attend to, so may end up missing some of the HIV-exposed children.* (Facility 1 IDI #1)

Nutritionist shortages also impact workflow. The inefficiency of *“go[ing] around the facility looking for a nutritionist to come and review the patient…who sometimes never comes”* was described by one respondent (Facility 2 FGD #3). Limited nutritionist availability because *“sometimes he has gone on strike, sometimes training, he is on leave”* (Facility 2 FGD #1) resulted in some patients not receiving nutritional screening or supplement prescriptions. One respondent elaborated,

*For example, you started your work today in the morning, your first client you say: 'Let me link to nutrition.' You send that client, then she comes back, 'There is no one.' [laughs] You will not send again the whole day.* (Facility 1 FGD #3)

While understaffing of nutritional services was one of the most frequently cited challenges, several participants noted that NGO-supported staffing in HIV services *“boosted”* service provision (Facility 1 FGD #2). NGO staffing support helped *“minimize waiting time”* and provided *“effective services”* (Facility 1 FGD #2).

### Inconsistency in material resource availability impacts retention in care and linkages between HIV and nutrition services

Health workers frequently described challenges due to an *“erratic”* supply of material resources (Facility 2 IDI #3), including antiretroviral drugs for infants, deworming drugs, immunization supplies, reagents for various lab tests including HIV, and nutritional supplements needed to treat malnutrition.

Health workers noted that reagent shortages mean that sometimes they are not able to offer HIV testing services for children experiencing malnutrition. One health worker explained that *“scarcity of [HIV] prophylaxis”* for infants caused *“some mothers [to] just stop breastfeeding immediately when they could not access prophylaxis because of that fear of their babies getting infected”* (Facility 1 FGD #1). Consequently, treatment shortages *“affected these babies’ growth because…they are being introduced into other foods at an early stage”* (Facility 1 FGD #1). During stockouts, health workers face challenges in providing advice on breastfeeding.

*[When there is a stock out] usually we advise the mother to take their medication if they are virally suppressed then the risk of transmission is always low, but…if the mother has a high viral load, and they are breastfeeding we go even to an extent of asking them to stop breastfeeding to cut off that risk of transmission.* (Facility 1 FGD #2)

Similarly, when nutritional supplements are out of stock guideline-adherent care cannot be provided to HEIs. One respondent explained: *“You might screen a child and find out that he or she meets the criteria for nutritional supplementation, but we don’t have the supplements”* (Facility 2 FGD #2). However, when nutritional supplements are in stock, patients might still not receive treatment and services due to understaffing (previously described). For example, health workers noted that relatives or caretakers sometimes are asked to prepare treatment feeds for the patient, without the expertise to do so, due to limited staffing at night.

Health workers proposed several solutions to overcome challenges in resource availability. They suggested increasing NGO support to supplement staffing and material resource gaps in nutrition and HIV services, such as providing *“formula feeds…for mothers who decide not to breastfeed up to six months”* (Facility 2 FGD #2). However, health workers also expressed concern about increasing NGO support, especially concerning what happens when partners cut funding or end programs. One health worker noted that “*on the part of the government mostly they are backed up by partners. So, sustainability, what if the partner wakes up and goes, you are back to square zero”* (Facility 2 FGD #3).

Other solutions proposed to counter erratic resource availability include continued communication between hospital administrators, the county, and the Kenya Medical Supply Authority to prioritize and strengthen investment in nutritional supplement supplies. Increasing nutrition-related knowledge of caregivers and families, particularly with *“male involvement,”* was also proposed to help families *“make nutritious food properly”* with *“what they have at home”* (Facility 1 FGD #3).

### Health workers have high intention to provide comprehensive HIV and nutritional care but low confidence that they have the resources to do so, damaging perceived self-efficacy and motivation

Health workers frequently expressed high intention to link children between HIV and nutritional care. Motivation to provide high-quality care to children was often driven by internal desire to help unwell children.

*We usually link [children between HIV and nutritional care] because you cannot see a child who has a problem and not link them because you also want the child to be well. So, in case a child is found to be sick, we must link the child to the nutritionist so that they can get help because we also don’t want to lose the child.* (Facility 1 IDI #3)

Health workers were also motivated by patient’s positive treatment outcomes, as one health worker explained,

*[Y]ou feel motivated to do better, if you realize that the linkage that you did, you cared for the child, you referred to a facility near and that person gives you a report about that child and you realize that the child is now doing well, you feel motivated and want to do more.* (Facility 2 IDI #1)

Conversely, poor outcomes, including child deaths, negatively impacted health workers’ motivation and job satisfaction. One respondent explained this impact: *“You know death will occur because if you don’t link, maybe you will lose a child or a mother, so it will affect [you]. Maybe you have tested ten, you linked two, eight are unlinked and then when you go for tracing you find 4 are dead. So, it will affect you”* (Facility 1 FGD #3).

However, health workers described minimal structured reinforcement to link patients between HIV and nutritional care, including limited direct supervision or incentives. Several respondents said health workers were not valued by their supervisors when they went out of their way to try to link nutritional care with pediatric HIV services and that *“most of the time supervisors only look at mistakes but going out of your way is never noticed”* (Facility 1 FGD #2). In contrast, some respondents said they did feel valued by their supervisors, despite lacking structured reinforcement. For example, one respondent said, *“Since I came to this facility I haven’t witnessed this but at the back of my mind I know there is someone who is seeing [my work] and maybe…if you are in the lower cadre they can promote you to a higher level”* (Facility 1 IDI #3).

The challenges health workers faced due to understaffing and erratic supply of material resources damaged perceived self-efficacy to link HEIs between services. Several health workers described feeling demoralized and demotivated by failures to link patients, particularly for nutrition care and treatment. One participant said,

*I am a nurse working in the pediatric ward…[M]y nutritionist I don’t know if he is on leave. I'm stuck with this patient, and I have no supplements to give. So, you're like, well, what are we even helping this patient with, we are just adding her more bill until the day that some nutritionist will come and see this child. Sometimes you feel like, what if I even had the number so that I call my nutritionist so that he can do that linkage because we feel it is very discouraging.* (Facility 2 FGD #1)

### Poverty makes it challenging for families to successfully link across HIV and nutritional services and follow treatment recommendations

Health workers described how poverty negatively affects HIV and nutrition-related outcomes for HEIs and their mothers, access to HIV and nutritional services, retention in care, and adherence to treatment.

*[T]he issue of poverty had also caused a big challenge at the community. [Facility 1] county poverty index, those who live below a dollar a day, the last study that was done we were around 74%, that is a big number…[Y]ou realize that however much you may try to educate these mothers on how they are supposed to feed these babies, some of them just fall into malnutrition.* (Facility 1 IDI #1)

Accessing care and follow-up for mothers and their infants can be difficult for families due to travel costs and distance from the health facility, even when *“the mother may be willing to go, but she doesn’t have the money”* (Facility 2 IDI #1). Several health workers also noted that prescriptions, hospital admission, and other treatment-related costs can be prohibitive and thus families do not attempt to access recommended HIV or nutritional services. While prescription drugs for children under five should be free, health workers reported that caregivers often have to buy supplies such as gloves, syringes, and medications. One respondent said that the high costs of treatment for families of malnourished HEIs can lead to patients leaving the health facility against medical advice, where they *“most likely…end up dying back in the community”* (Facility 2 FGD #1). Health workers reported that some families share nutritional supplements amongst multiple children, resulting in lack of improvement for the intended patient. To address poverty in the community, health workers proposed implementing income generating activities for economic empowerment of the families and to improve patient nutritional status.

### The double burden of HIV and malnutrition-related stigma makes it challenging for families to link between HIV and nutritional care

HIV and malnutrition-related stigma was commonly reported by respondents as a barrier to care. Health workers said that some caregivers living with HIV might leave the clinic out of fear of HIV status disclosure when familiar community members are present. However, the physical integration of MCH services was cited as a potential mitigator of stigma for caregivers because they can get *“every service…within the same premises”* (Facility 2 FGD #3). Additionally, stigma negatively affected adherence to antiretroviral regimens for infants recently diagnosed with HIV if the mother’s HIV status was not known to her family. For some caregivers, infant malnutrition is also stigmatizing. As one health worker explained,

*[S]tigma is also a challenge, we see so much in the wards sometimes if you talk to the parents and you are trying to tell them that what the child is suffering from is malnutrition, they really try to argue that how come, that they are feeding, they are giving food…And no one would ever want to be associated with malnutrition because to them somehow it is poverty, is it like the nurse who is talking to me, how have they perceived me.* (Facility 2 IDI #1)

Several health workers described that nutritional supplements are perceived as *“things meant for HIV positive people”* (Facility 1 IDI #3). Fear of being stigmatized by community members or outed to family members, including husbands, as living with HIV can lead some caregivers to reject prescription of supplements, *“leave [supplements] on the road”* (Facility 2 FGD #1), or *“pour [supplements] out and use the packaging to start her charcoal stove”* (Facility 1 IDI #3).

Health workers noted ongoing efforts to reduce stigma and strengthen privacy for caregivers living with HIV and their HEIs, including support groups for mothers living with HIV to *“know that ‘I am not alone, we are many’”* (Facility 2 IDI #1), and protecting mothers’ privacy and confidentiality during clinic visits. Other suggestions to address stigma were to strengthen disclosure procedures to aid clients in *“accept[ing] the status”* (Facility 1 FGD #2) and create support groups and clinic days for adolescent mothers living with HIV who might face increased stigma.

## Discussion

This study identified determinants of guideline-adherent delivery of coordinated HIV and nutrition care to HEIs in western Kenya. Findings highlight the interaction between patient-, provider-, and system-level factors that contribute to provider deviation from national HIV and nutritional guidelines for HEIs. Findings underscore existing evidence that shortages of human and material resources [30], family resources [31], and stigma impact care for HEIs and children with malnutrition. This study also includes novel findings regarding the impact of resource constraints, the promise of supportive supervision for improving staff motivation, the need for enhancing collaboration between health system actors, and ideas for improving patient referral processes for HEIs and malnourished children.

We utilized the TDF as a guiding framework and found that three domains exhibited greatest influence on guideline adherent care: environmental context and resources, beliefs about capabilities, and social influences. This finding is consistent with the domains identified in other applications of the TDF to identify drivers of guideline adherence. A study that used the TDF to understand adherence to primary care guidelines in South Africa similarly found that aspects of the environmental context were a barrier to implementation, even while motivation to use the guidelines was high [32]. Meanwhile, when implementing pediatric treatment guidelines in Laos, health providers highlighted the social influences of their colleagues as well as preferences of patient families as factors influencing guideline adherence [33].

There was consensus among health workers in this study that resource gaps (time, personnel, supplies) make it challenging to adhere to guidelines for HEIs, even when guideline knowledge is high. Our findings are consistent with evidence that system-level resource challenges are closely linked with health worker guideline adherence, both by directly limiting opportunities to adhere to guidelines and by impacting the self-efficacy and motivation of health workers [34]. Successful implementation of guidelines occurs through a process of normalization, during which they become part of the standard practice of health workers [35]. When resource availability is erratic, it is especially difficult to establish these consistent routines that align with guidelines.

Two interventions that may address identified challenges in adhering to guidelines for HEIs are task shifting and group-based counseling. In some settings in Kenya where nutritionists are scarce, task shifting to breastfeeding peer supporters has enhanced nutrition services for infants [36]. Offering PMTCT and nutrition care to clients in groups may also enable limited staff to reach more clients; these models have lowered costs and produced equivalent or better outcomes in antenatal care [37] and antiretroviral therapy services, including among post-partum women [38, 39]. Moreover, capacity development may be needed to ensure health workers are competent to deliver relevant and structured nutrition counseling messages to caregivers of HEIs [40].

Enhancing supportive supervision for staff is a promising approach that may improve linkages between HIV and nutritional care by addressing staff motivation. In this study, health workers reported that supervision was infrequent and typically focused on mistakes. There is evidence that health worker motivation is negatively impacted by low levels of organizational support, time pressure, high workload, unavailability of resources, and stress, which can increase burnout in health facilities in low- and middle-income countries [41]. A study in Kenyan district hospitals found that supportive hospital leadership and effective teamwork across cadres are among the most important drivers of health worker motivation [42].

In many settings, such as the two facilities included in this study, PMTCT staff are managed by donor-funded implementing partners and nutrition staff through the government health system. While participants reported good teamwork among staff managed by both programs, frontline health workers also identified differences in training opportunities, role expectations, and staff supervision between programs that resulted in feeling “segregated.” PMTCT staff managed by partners had more training opportunities, leaving other staff feeling less qualified to perform certain activities (e.g., breastfeeding counseling for mothers with HIV, malnutrition treatment). Strengthening constructive collaboration between actors in the health system is critical to fully integrating HIV and nutrition services and improving service delivery [43].

### Strengths and limitations

Preliminary themes were validated by sharing them with health workers at study sites using a process known as member checking [29]; participants confirmed that the preliminary themes aligned with their experiences and captured key barriers and facilitators they face when delivering care. While health workers described patient-level determinants to care (e.g., stigma), we did not interview patient caregivers directly and thus these findings are understood through the lens of facility staff. Additionally, the experiences of providers based at two county referral hospitals in western Kenya may not be generalizable to other regions or types of facilities.

## Conclusion

Understanding health worker experiences providing HIV and nutrition services to HEIs can inform efforts to identify potential solutions and improve guideline-adherent care. This study confirmed that facilitators and barriers to guideline-adherent care occur across system-, provider-, and patient-levels. For example, human and material resource constraints frequently underpinned barriers to successful linkages. In addition, the poverty and stigma faced by patients and their families impacted care, despite the best efforts of health workers with adequate knowledge of care guidelines. This study expands upon existing evidence by highlighting the benefits of physical integration of service sites and the impact of system challenges on the motivation of individual health workers. Health workers identified opportunities to address these challenges by improving access to needed resources, providing additional training, task sharing, and increasing supportive supervision. Future implementation research should focus on the scale up and sustainment of interventions that address these determinants of guideline-adherent nutritional care for HEIs.

## Supplementary Information

Supplementary Material 1.

## Data Availability

Transcripts of IDIs and FGDs are not available, as the information provided may be considered identifiable. Case memos developed during data analysis (redacted to ensure anonymity) will be available from the corresponding author upon reasonable request.

## Abbreviations

FGD: Focus Group Discussion
HBCTRH: Homa Bay County Teaching and Referral Hospital
HEI: HIV-Exposed Infant
HIV: Human Immunodeficiency Virus
IDI: In-Depth Interview
MCH: Maternal Child Health
MCRH: Migori County Referral Hospital
MOH: Ministry of Health
NGO: Non-Governmental Organization
PMTCT: Prevention of Mother to Child Transmission
TDF: Theoretical Domains Framework
