# Supplementary material for: Factors influencing provider deviation from national HIV and nutritional guidelines for HIV-exposed children in western Kenya: a qualitative study
Source: BMC Health Serv Res. 2024 Nov 26;24:1473. doi: 10.1186/s12913-024-11942-9 (PMC11600710; doi:10.1186/s12913-024-11942-9)
Supplement: Supplementary file 1 — Supplementary Material 1. [file 12913_2024_11942_MOESM1_ESM.docx]

## **Appendix 1**

**Question Guide**

For Focus Group Discussions and Interviews

**Facilitator welcome message:** Thank you for joining this focus group discussion. A warm welcome to you all. The purpose of this discussion is to learn about how HIV early infant diagnosis and nutritional services are provided together or separately to young children at this facility. As you know, children who are exposed to HIV are very vulnerable to malnutrition, and other diseases. And there can be many things that make it easier or harder to provide nutritional screening and care for these children. In this conversation, the goal is to learn about your experiences linking HIV and nutritional care for young children. We will discuss what is working well, what challenges you face, and discuss ideas for how we might overcome those challenges. Do you have any questions about the purpose of this discussion, before we begin?

1. I would like to start by discussing what services are currently provided at the health facility. Tell me about the nutritional services that HIV-exposed children currently receive, if any.
   1. *Probe:* What are the specific services, and who provides them?
2. What nutritional monitoring or care are children involved in early infant diagnosis programs supposed to receive at your health facility?
   1. *Probe*: Do we all agree on this list of services?
   2. *Probe*: If not, why not?
3. What HIV screening or other services are children involved in outpatient nutrition clinics intended to receive?
   1. *Probe*: Do we all agree on this list of services?
   2. *Probe*: If not, why not?
4. How challenging is it to deliver nutritional screening and care to all HIV-exposed children in your health facility?
5. What factors make it possible to deliver nutritional care to HIV-exposed children in the health facility?
   1. *Probe*: These factors might be related to issues such as guideline clarity, access to material resources, facility processes, leadership, human resources, or any number of other issues. Do you have anything else to add?
6. We have talked about factors that make it harder and easier to deliver nutritional care to HIV-exposed children. What factors make it harder or easier to link children seen in nutrition clinic to early infant diagnosis services for HIV testing?
   1. Are these issues related to family preferences and stigma?
   2. Are these issues related to clinic procedures or norms?
7. What solutions do you think would help you overcome those challenges?
   1. *Probe:* Are additional resources necessary to overcome those challenges?
   2. *Probe:* Do health workers require any new skills to overcome those challenges?
   3. *Probe:* Are any new trainings needed to develop those skills?
8. Do most of the health workers and staff at the facility who are involved in pediatric care try to link HIV and nutritional services? Why or why not?
9. Do you feel it is valued by peers or supervisors when health workers go out of their way to try to link nutritional care with pediatric HIV services? Is it important to health workers? Why or why not?
10. We have discussed many aspects of nutritional and HIV services at the health facility, and the ways in which they are or are not linked to one another. How do these linkages, or the lack of linkages, affect your personal motivation or satisfaction with your job?

**Facilitator: I am now going to ask you some questions about what steps could be taken to improve the way that nutritional services are provided to HIV-exposed children, or that HIV and nutritional care are linked more generally.**

1. What would an ideal program look like that was able to meet the nutritional needs of all HIV-exposed children in your health facility?
   1. *Probe*: What services would be provided? By whom? And where?
   2. *Probe****:*** What improvements could be made now to start working towards this improved system?
   3. *Probe***:** Is there anything that you personally can do to make these changes? Are these changes possible to make? Why or why not?
2. If these changes that you have proposed were made, and nutrition and early infant diagnosis programs were more efficiently delivered together, what would the outcomes be?
   1. *Probe:* What might be the outcomes for children and their families?
   2. *Probe:* What might the outcomes be for you and your colleagues in terms of your daily work activities?
   3. *Probe:* Would anything change at the health facility-level, how so?
3. Do you think improving nutritional care for HIV-exposed children is a priority for the health facility?
   1. *Probe*: Why or why not?
   2. *Probe*: Do you
4. Is there anything else related to linking HIV and nutritional care for children that you feel is important to discuss or share?

## **Appendix 2**

**Lishe Bora Qualitative Codebook**

| **Domain / *Construct^1^*** | **Domain / Construct Definition** | **Inclusion/Exclusion for Code Application** | **Code** |
| --- | --- | --- | --- |
| Knowledge | An awareness of the existence of something | **Inclusion Criteria:**  Include statements regarding knowledge/lack of knowledge of nutritional and HIV care for HIV-exposed infants.  **Exclusion Criteria:**  Exclude statements referring to skills health care workers use to deliver HIV or nutritional care. Skills should be applied to excerpts that are action oriented or verb-based. | Knowledge |
| Skills | An ability or proficiency acquired through practice | **Inclusion Criteria:**  Include statements regarding skills health workers need to deliver nutritional or HIV care to children.  **Exclusion Criteria:**  Exclude statements regarding knowledge as it relates to service provision. | Skills |
| *Skills development* | Process of identifying, developing, and honing skills | **Inclusion Criteria:**  Include statements regarding how health workers initially develop or improve their skills needed to deliver nutritional or HIV care to children. | Skills_development |
| *Ability* | Having the means or skills to complete a task | **Inclusion Criteria:**  Include statements regarding whether health workers have the ability to deliver nutritional or HIV care to children. For example, “Nurses cannot do XYZ.”  **Exclusion Criteria:**  Exclude statements regarding self-efficacy. For example: “I don’t think nurses are capable of doing XYZ.” Self-efficacy refers to whether individuals *perceive* that they have the capabilities to do XYZ, while ability refers to whether an individual *can* *do* XYZ. | Skills_ability |
| *Practice* | Repetition of an act, behavior, or series of activities to improve a performance or develop a skill | **Inclusion Criteria:**  Include statements regarding if and how health workers practice to improve their skills needed to deliver HIV and nutritional care to children. | Skills_practice |
| Social/professional role and identity | A coherent set of behaviors and displayed personal qualities of an individual in a social or work setting | **Inclusion Criteria:**  Include statements regarding health workers’ roles and responsibilities in providing HIV and nutritional care. Include statements regarding how different roles interact with each other (e.g., teamwork).  **Exclusion Criteria:**  Exclude statements that do not specifically state what role is carrying out the responsibility. | Role_identity |
| *Leadership* | Act of leading a group or organization to accomplish group or organizational goals | **Inclusion Criteria:**  Include statements regarding health workers’ role in leadership within the facility. Also include statements regarding the role of facility leadership and health workers’ relationship/interaction with facility leadership. | Role_identity_  leadership |
| *Organizational commitment* | Dedication and connection employees have with their organization | **Inclusion Criteria:**  Include statements regarding whether health workers feel committed to the facility they work in and its goals/objectives. | Role_identity_  organizational |
| Beliefs about capabilities | Acceptance of the truth, reality or validity about an ability, talent or facility that a person can put to constructive use | **Inclusion Criteria:**  Include statements regarding beliefs in ability to implement high quality nutritional and HIV care systems, improve delivery, or follow treatment and care plans. | Beliefs_capability |
| *Self-efficacy* | Individual's belief in his or her capacity to execute behaviors necessary to produce specific performance attainments | **Inclusion Criteria:**  Include statements regarding beliefs in ability to complete the necessary actions required to provide high-quality HIV and nutritional care to children or follow treatment and care plans. | Beliefs_capability_  self_efficacy |
| *Perceived behavioral control* | Individual’s perception of the difficulty of enacting a behavior | **Inclusion Criteria:**  Include statements regarding how easy or difficult individuals perceive adopting new skills, systems, or behaviors to improve, implement, or follow HIV and nutritional care treatment and care. | Beliefs_capability_  behavioral_control |
| *Empowerment* | The progression of choice, influence, and control an individual can exercise over events in their lives | **Inclusion Criteria:**  Include statements regarding if and/or how individuals believe they have choice, influence, and control over providing or receiving high-quality HIV and nutritional care for children. | Beliefs_capability_  empowerment |
| Optimism | The confidence that things will happen for the best or that desired goals will be attained | **Inclusion Criteria:**  Include statements related to whether HIV and nutritional care can and will be improved. | Optimism |
| Beliefs about consequences | Acceptance of the truth, reality, or validity about outcomes of a behavior in a given situation | **Inclusion Criteria:**  Include statements regarding perceived consequences of successfully linking or of not linking children to nutritional and HIV care. Include statements regarding what consequences on a patient-, health worker-, and facility-level individuals see and/or experience as a result of providing/receiving or not providing/receiving HIV and nutritional care for children. | Beliefs_consequences |
| *Anticipated regret* | The sense of potential regret an individual may feel in the future that influences their decision making | **Inclusion Criteria:**  Include statements regarding regret individuals may think they will feel (or not feel) in the future related to how HIV and nutritional care is delivered to children. | Beliefs_consequences  _regret |
| *Positive consequences* | Positive results, experiences, emotions, or attitudes that result from a given situation | **Inclusion Criteria:**  Include statements regarding positive perceived or experienced consequences from the successful linkage and delivery of HIV and nutritional care of children. Include statements regarding motivation as a result of delivery of care and successful linkage of services.  **Exclusion Criteria:**  Exclude statements regarding reinforcements, which specify an arranged system of incentives or punishment to encourage a specific behavior. | Beliefs_consequences_  positive |
| Reinforcement | Increasing the probability of a response by arranging a dependent relationship, or contingency, between the response and a given stimulus | **Inclusion Criteria:**  Include statements regarding general reinforcements (i.e., arranged rewards) for individuals to provide or receive high-quality HIV and nutrition care for children.  **Exclusion Criteria:**  Exclude statements regarding perceived or actual consequences from a given situation or stimulus. Code these statements using “Beliefs about consequences.” | Reinforcement |
| *Incentives* | Any stimulus that encourages a desired response | **Inclusion Criteria:**  Include statements on motivators (i.e., arranged incentives) for providing or receiving HIV and nutritional care. | Reinforcement  _incentives |
| *Punishment* | Any penalty as a consequence of an undesirable action or outcome | **Inclusion Criteria:**  Include statements regarding supervision and punishment or consequences of health worker behaviors. | Reinforcement_  punishment |
| Intentions | A conscious decision to perform a behavior or a resolve to act in a certain way | **Inclusion Criteria:**  Include statements regarding whether and how much health workers involved in pediatric care try to link HIV and nutritional services for children, and why intentions may be high or low. | Intentions |
| Goals | Mental representations of outcomes or end states that an individual wants to achieve | **Inclusion Criteria:**  Include statements on what an ideal program looks like to meet the nutritional needs of all HIV-exposed children. Include related statements on outcomes of an ideal program for children, families, health workers, and facilities. | Goals |
| *Goal priority* | Order of importance or  urgency of goals | **Inclusion Criteria:**  Include statements regarding order of importance of goals related to improving HIV and nutritional care for children. | Goals_priority |
| Memory, attention, and decision processes | The ability to retain information, focus selectively on aspects of the environment and/or choose between two or more alternatives | **Inclusion Criteria:**  Include statements regarding making nutritional and HIV care delivery decision making. | Processes |
| Environmental context and resources | Any circumstance of a person’s situation or environment that discourages or encourages the development of skills and abilities, independence, social competence and adaptive behavior | **Inclusion Criteria:**  Include general statements regarding environmental context (e.g. facility-level infrastructure and resources) that impacts health workers’ ability to provide HIV or nutritional care. | Environment |
| *Resources/material resources* | Availability of human and material resources | **Inclusion Criteria:**  Include statements related to human and material resource availability (e.g., staff, nutrition supplements, MUAC tape) that make it easier or harder to deliver integrated HIV and nutrition services to children. | Environment_  resources |
| *Organizational culture/climate* | Any aspect related to the organizations working culture or climate | **Inclusion Criteria:**  Include statements regarding health facilities’ organizational culture/climate, including how organizational culture may make it easier or harder to deliver integrated HIV and nutritional services to children. | Environment_  organization |
| *Systems, processes, and group norms* | Procedures according to which something is done | **Inclusion Criteria:**  Include statements regarding formal and informal systems, procedures, and processes involved in the delivery of HIV and nutritional care to children. Include group norms related to these systems, procedures, and processes. | Environment_systems |
| Social influences | Interpersonal processes that can cause individuals to change their thoughts, feelings, or behaviors | **Inclusion Criteria:**  Include statements regarding group norms, family preferences, religious beliefs, and/or social stigma impacting HIV and nutritional care delivery. | Social_influences |
| *Modelling* | The process in which one or more individuals serve as examples for another individual to copy | **Inclusion Criteria:**  Include statements regarding learning how to deliver HIV and nutritional care to children from other health workers via modelling. | Social_influences  _modelling |
| *Social support* | The provision of assistance to others, typically in order to help them cope with stressors | **Inclusion Criteria:**  Include statements regarding how social support is given or received before, during, or after HIV or nutritional care for children. | Social_influences  _social_support |
| Emotion | A complex reaction pattern, involving experiential, behavioral, and physiological elements, by which the individual attempts to deal with a personally significant matter or event | **Inclusion Criteria:**  Include statements related to mental health, burn-out, and/or positive or negative affect when delivering or receiving care. | Emotion |
| Behavioral regulation | Anything aimed at managing or changing objectively observed or measured actions | **Inclusion Criteria:**  Include statements regarding how health workers are planning to change, act, and/or manage improvements to delivery of HIV and nutritional care. Include statements related to changing facility norms to improve delivery of care.  **Exclusion Criteria:**  Exclude statements regarding intentions. Code these statements as ‘Intentions.’ Note intention is about the decision to perform a behavior, whereas behavioral regulation is about actions individuals take to try to change their behavior. | Behavioral  _regulation |
| Patient and family beliefs and behaviors | Any attitudes, knowledge, or actions by patient, family, or community members | **Inclusion Criteria:**  Include statements describing patient, family, and community member beliefs, attitudes, knowledge, and/or behaviors.  **Exclusion Criteria:**  Exclude statements regarding health worker beliefs, attitudes, knowledge, and/or behaviors. As default, all statements without this code will be assumed as pertaining to health workers. | Patient_family |
| Good quotes | Exemplary excerpts or statements that stand out as potentially important references for analysis | **Inclusion Criteria:**  Include statements that stand out as particularly descriptive, insightful, or exemplary regarding delivery of HIV and nutritional care for children. | Good_quotes |

^1^ Constructs included in the codebook will only be coded as secondary “child codes”. Coders could code first with the parent codes (domains). However, if a child code provides relevant elaboration upon the parent code, it should be coded as well
